# Perceptions of undergraduate medical students on artificial intelligence in medicine: mixed-methods survey study from Palestine

**DOI:** 10.1186/s12909-024-05465-4

**Published:** 2024-05-07

**Authors:** Kamel Jebreen, Eqbal Radwan, Wafa Kammoun-Rebai, Etimad Alattar, Afnan Radwan, Walaa Safi, Walaa Radwan, Mohammed Alajez

**Affiliations:** 1 Department of Mathematics, Palestine Technical University – Kadoorie, Hebron, Palestine; 2 Department of Mathematics, An-Najah National University, Nablus, Palestine; 3 Unité de Recherche Clinique Saint-Louis Fernand-Widal Lariboisière, APHP, Paris, France; 4 Department of Biology, Faculty of Science, Islamic University of Gaza, Gaza, Palestine; 5 Medical Research Direction, Ministry of Health, Tunis, Tunisia; 6 Faculty of Education, Islamic University of Gaza, Gaza, Palestine; 7 Department of Biotechnology, Faculty of Science, Islamic University of Gaza, Gaza, Palestine; 8 University College of Applied Sciences - Gaza, Gaza, Palestine; 9 Al-Azhar University, Gaza, Palestine

**Keywords:** Artificial intelligence, Medical students, Medical education, Machine learning, Perceptions, Cross-sectional study, Palestine

## Abstract

**Background:**

The current applications of artificial intelligence (AI) in medicine continue to attract the attention of medical students. This study aimed to identify undergraduate medical students’ attitudes toward AI in medicine, explore present AI-related training opportunities, investigate the need for AI inclusion in medical curricula, and determine preferred methods for teaching AI curricula.

**Methods:**

This study uses a mixed-method cross-sectional design, including a quantitative study and a qualitative study, targeting Palestinian undergraduate medical students in the academic year 2022–2023. In the quantitative part, we recruited a convenience sample of undergraduate medical students from universities in Palestine from June 15, 2022, to May 30, 2023. We collected data by using an online, well-structured, and self-administered questionnaire with 49 items. In the qualitative part, 15 undergraduate medical students were interviewed by trained researchers. Descriptive statistics and an inductive content analysis approach were used to analyze quantitative and qualitative data, respectively.

**Results:**

From a total of 371 invitations sent, 362 responses were received (response rate = 97.5%), and 349 were included in the analysis. The mean age of participants was 20.38 ± 1.97, with 40.11% (140) in their second year of medical school. Most participants (268, 76.79%) did not receive formal education on AI before or during medical study. About two-thirds of students strongly agreed or agreed that AI would become common in the future (67.9%, 237) and would revolutionize medical fields (68.7%, 240). Participants stated that they had not previously acquired training in the use of AI in medicine during formal medical education (260, 74.5%), confirming a dire need to include AI training in medical curricula (247, 70.8%). Most participants (264, 75.7%) think that learning opportunities for AI in medicine have not been adequate; therefore, it is very important to study more about employing AI in medicine (228, 65.3%). Male students (3.15 ± 0.87) had higher perception scores than female students (2.81 ± 0.86) (*p* < 0.001). The main themes that resulted from the qualitative analysis of the interview questions were an absence of AI learning opportunities, the necessity of including AI in medical curricula, optimism towards the future of AI in medicine, and expected challenges related to AI in medical fields.

**Conclusion:**

Medical students lack access to educational opportunities for AI in medicine; therefore, AI should be included in formal medical curricula in Palestine.

**Supplementary Information:**

The online version contains supplementary material available at 10.1186/s12909-024-05465-4.

## Introduction

The health systems in the world have undergone major changes with the incorporation of technological advances, particularly in the post-COVID-19 pandemic era [1]. Artificial intelligence (AI) has attracted the interest of specialists and professionals in medicine in recent years because of its extensive medical applications [2]. AI has the potential to be a helpful medical tool in diagnostics and clinical decision-making due to its excellent capacity to integrate vast volumes of clinical data [3, 4]. AI applications in medicine have the potential to support a variety of tasks such as clinical research [5], therapy [6], administrative procedures [7], and drug development [8] due to their capacity to learn from large sets of clinical data.

Globally, numerous countries have begun implementing AI to enhance the effectiveness of their healthcare system delivery [9]. Many clinicians and specialists in the medical field are expecting the use of AI, machine learning (ML), neural networks (NN), and deep learning (DL) in diagnostics, prognostics, and treatment [10–14]. However, as the adoption of AI systems spreads in the medical sector, issues concerning the ethical ramifications of employing this technology become increasingly important [15, 16]. As evidenced by the COVID-19 pandemic, AI has an impact on medical education where it provides medical students with an interactive learning environment and creates virtual simulations and training, allowing them to practice complicated procedures on virtual patients without endangering actual patients [17–20]. However, due to a lack of teachers qualified in AI, the expensive cost of AI software, and ethical issues, many educational institutions struggle to effectively integrate AI into their teaching practices [21, 22]. The introduction of ChatGPT-4, a new AI-driven language model, revealed its potential for helping students understand difficult scientific lessons, but it also highlighted ethical issues [23].

With AI applications poised to have a significant influence on medical practices, attention is being drawn to how the medical workforce can be ready for this transition and, thus, to investigating the perceptions of medical and healthcare professionals, or medical students [24–34]. Many studies investigated the knowledge, perception, and attitudes of AI for medical and healthcare professionals or medical students in countries such as the Republic of Korea [24], Germany [25, 26], the UK [27, 28], Canada [29], the USA [30], Pakistan [31], Australia and New Zealand [32], Malaysia [33], Turkey [34], Saudi Arabia [35], Egypt [36], Syria [37], the United Arab Emirate [38], and Kuwait [39].

An evident knowledge gap is present in this field in Palestine, which must be addressed due to the inevitable future increase in AI use in the medical field. It is important to understand the attitudes and behaviors of medical students as end users of AI applications in the future to integrate AI into medicine and medical education. Additionally, evaluating students’ perceptions of AI is essential to determining whether additional training may later be needed, given that they will constantly interact with patients and use technology. However, based on our knowledge, there are no recent studies investigating the perceptions of Palestinian medical students about the integration of AI in medical education. Therefore, this study aims to identify undergraduate medical students’ attitudes toward AI in medicine, explore present AI-related training opportunities, investigate the need for AI inclusion in medical curricula, and determine preferred methods for teaching AI curricula. The findings will help in making decisions about medical AI implementation and the development of medical curricula in the future.

## Methods

### Study design

This study was prepared according to the Checklist for Reporting of Survey Studies (CROSS) (Supplementary File 1). This descriptive, cross-sectional, mixed-methods, questionnaire-based study was conducted in the West Bank and Gaza Strip, Palestine, between June 15, 2022, and May 30, 2023. A mixed-methods design was selected to allow researchers to gather data from a large sample size using a survey while also providing a more comprehensive investigation and a narrative context through interviews. Interviews are considered helpful tools in examining institutionally specific potential or limitations that the survey cannot determine, given how different medical curricula might be across universities. The target population was undergraduate medical students who enrolled in the faculty of medicine at a private or public university in Palestine in the academic year 2022–2023.

### Phase one: quantitative study

#### The questionnaire

We developed the questionnaire dimensions and questions from the survey designed by Pucchio et al. [40], who validated the survey through a study of the literature and discussions with medical professionals and health experts. The changes in relation to participants’ characteristics and AI questions were drawn from published articles and literature reviews [41, 42]. We modified the items and questions extracted from previous studies to fit the Palestinian medical students. The questionnaire was applied in English, which is the language of medical education in Palestine.

The questionnaire was divided into five sections (Supplementary File 2) with 49 questions. The first section consisted of screening questions to exclude participants who did not match the requirements for inclusion (4 questions). The second section included questions regarding demographic characteristics (7 questions). The third and fourth sections inquired the study participants about their knowledge of AI in daily life (6 questions) and medicine (19 questions), respectively. The final section asked participants about their preferred learning methods for AI as well as their educational opportunities about AI during their medical education or training (13 questions). The Cronbach’s alpha coefficient was found to be 0.891, indicating high internal consistency.

### Pilot study

The questionnaire was piloted before distribution to test the viability of the questions, clarify misunderstood sentences, identify any unclear questions, and test the accessibility of online forms. Taking the sample size into consideration, the estimation was done using the probability equation and confidence interval. Viechtbauer et al. [43] provided a good strategy for choosing a sample size for a pilot study that ensures high confidence in detecting potential difficulties. The confidence level for our calculations is set at 95%, which corresponds to a significance level of 5%. The equation for sample size sampling is Eq. (1), as follows:

1 $${\rm{n}}\,{\rm{ = }}\,{\rm{ln}}\,{\rm{(1 - }}\gamma {\rm{)}}\,{\rm{/}}\,{\rm{ln}}\,{\rm{(1 - }}\pi {\rm{)}}$$

where.

n = sample size.

π = Problem probability or significance level = 0.05.

y = Level of confidence = 0.95.

To ensure that the problem was identified with a high degree of confidence, n = ln (1-0.95)/ln (1-0.05) = 58.40, or 59 participants, had to be included in the pilot study. The pilot study was conducted separately, and the findings were not used in the current study. Following the completion of the pilot study, the survey was administered to undergraduate medical students in Palestine.

### Sample size calculation

We recruited the study participants by a nonprobability convenience sampling among medical students who study at Palestinian universities. In convenience sampling, the participants are selected based on availability and willingness to take part and this is suitable to the study (Wang and Cheng, 2020). Based on a 5% margin of error, a 95% confidence level, and a 50% outcome response, the estimated sample size was 371 students, where the population was 10,361 students (Table 1). This sample size is believed to be adequate for the study’s target population. The researchers invited 371 undergraduate medical students to complete the questionnaire. All undergraduate medical students are studying in private or public universities, which are supervised by the Palestinian Ministry of Education.

The criteria for selecting the participants were as follows:

1. Students who are currently enrolled in the faculty of medicine at a private or public university in Palestine.
2. Undergraduate medical students in any year of study.
3. Medical students who have temporarily stopped studying medicine (postponement of studies due to economic, social, or health conditions).
4. Undergraduate students who transferred from the faculty of medicine to other faculties (such as the Faculty of Science, Health Sciences, Pharmacy, or Nurse) and studied for at least one year in the faculty of medicine.
5. Students of Palestinian nationality.
6. Students residing in Palestine (the West Bank or Gaza Strip).

Criteria for excluding participants:

1. Medical students who graduated from the university and finished their medical studies.
2. Medical students who left their studies.
3. Undergraduate students who transferred from the faculty of medicine to other faculties (such as the Faculty of Science, Health Sciences, Pharmacy, or Nurse) and studied only one semester in the faculty of medicine.

**Table 1:** Distribution of undergraduate medical students by universities in Palestine

University	New students	Enrolled students	Total
Al-Azhar University	465	1258	1723
Islamic University of Gaza	230	964	1194
Hebron University	155	478	633
Palestine Polytechnic University	227	725	952
Al-Quds University	619	2056	2675
An-Najah National University	746	1911	2657
The Arab American University	162	365	527
**Total**	**2604**	**7757**	**10,361**

(Source: Palestinian Ministry of Higher Education, https://www.mohe.pna.ps/services/statistics)

### Questionnaire distribution

The participants were informed about the inclusion criteria, which were written on the title page of the questionnaire. To ensure participant privacy, all responses were anonymously collected. Emails and phone numbers were only collected for later contact with participants who agreed to participate in the interview part of the study. We used Google Forms to create an electronic questionnaire version. Participation was completely voluntary, and no rewards or incentives were provided for participation. After getting informed consent from the students, they were asked to answer the following question: “Are you willing to participate in this study?“. If the participants selected “yes,” they were given access to the questionnaire.

The invitations to participate and the questionnaire were sent to medical students’ accounts on medical private groups with the help of colleagues, professors, and academic staff databases. To ensure that only medical students fill out the questionnaire, we sent the link to medical groups (Facebook, WhatsApp, and Telegram), which has been created by the faculty members. These groups have been created to contact students concerning sending, receiving, uploading, and downloading medical materials and advertising any scientific events. These groups included only medical students who registered in a specific course (molecular biology, physiology, parasitology), and the administrators in these groups only accepted students after confirming the presence of their name on the registered list. The invitation link included inclusion criteria, a brief background, information on the study’s protocols, goals, voluntary participation, assurances of anonymity and confidentiality, and a link to the questionnaire.

We prevented duplication of respondents by email address. We did not allow access to the questionnaire without an email address, so the participants had only one access to fill out the questionnaire. Also, the statistician checked out the email address list to ensure that there were no duplicate responses from the same participant.

To conduct the interviews, the researchers contacted a representative random sample of medical students who answered “yes” to the question, “Would you like to participate in an interview that will be conducted by the researchers?”.

### Phase two: qualitative study

#### Sampling method and interview setting

A group of participants underwent follow-up interviews to gain further information about educational opportunities regarding AI that were not covered in the questionnaire. Purposive sampling was carried out at this stage. The interviews were conducted in compliance with the standards for reporting qualitative research set by the Consolidated Criteria for Reporting Qualitative Research (COREQ) [44]. Participants who were willing to participate in the second stage of the study were invited to the interview, which was conducted by two trained researchers. The interviews were conducted via Google Meet for 5–20 min. The interview consisted of a set of predetermined, open-ended questions covering their attitudes towards AI as well as their opinions on getting an AI education during their study or training (Supplementary File 3). The questions from the interviews were extracted from a previous survey [40]. The interview questions aimed to provide information for upcoming studies and the creation of medical curricula about AI in medicine. During the interview, the researcher recorded the responses for analysis.

### Designed interview protocol

The interviews were conducted by trained researchers (ER and AR). The objectives of the study were explained at the beginning of each interview, and verbal consent was obtained from each participant. The participants were provided with essential background information. There were no extra questions asked after the interviews, no additional information was given by the interviewers, and no other questions were requested subsequently. Only the interviewers and the interviewee were present for the interview. Other individuals were not allowed to attend the interview with the participant to ensure the privacy and confidentiality of the response. Before recording the responses, permission for recording was requested by the researcher.

In the current study, fifteen undergraduate medical students were included in the interviews. Females constituted 60.0% (9) of the total interviewed participants. Four participants were in their first year of medical school, six were in their second year, one was in their third year, and four were in their fifth year. The objectives of the study were all well understood by the participants.

### Data collection

Data was gathered using individual, in-depth interviews. The research team analyzed the study questions and methods for collecting reliable data before starting the interviews. During the interviews, the required data were recorded through note-taking. The collection of data was continued until it reached saturation and no new topics started in the interviews.

### Statistical analysis

The data were analyzed in R-Cran version 4.3.0. Descriptive statistics and inductive thematic analysis were used to analyze quantitative and qualitative data, respectively. Data are presented as means and standard deviations (SD) for normally distributed continuous variables; as medians and interquartile ranges (IQR) for non-normally distributed numeric variables; and as n (%) for categorical data. Mann-Whitney U or Kruskal-Wallis H tests were used to verify differences in the variables based on demographic differences. *P* values < 0.05 were considered statistically significant. According to the Likert scale, the frequency of pertinent responses and the proportion of participants who agreed or disagreed with each item were reported. The data was excluded from participants who did not meet the required answers in the screening questions or did not respond to all questions of the questionnaire. Internal consistency was determined using Cronbach’s alpha. The Ggplot package (version 3.4.2) on R-Cran version 4.3.0 was used to design a stacked bar plot of Likert scale data.

To investigate the experiences of medical students with AI in medicine and their potential need for AI in medical education, a qualitative inductive content analysis approach was used to examine the text transcriptions for each sentence separately. We carefully read text transcriptions line by line and then coded for each incident by detecting participants’ exact words without interpretations. All responses were initially coded inductively for the main themes. The next step was to sort codes according to similar meanings and identify the clustering of codes, which formed subthemes. The subthemes were sorted according to similar meanings and named to fit the cluster. These procedures have been achieved manually. To verify the rigor of this study, we used Lincoln and Gube’s [45] criteria for confirmability, credibility, dependability, and transferability. There were no missing data.

### Ethics approval

The present study was conducted according to the guidelines of the Declaration of Helsinki and approved by the Ethics Committee of the Faculty of Science of the Palestine Technical University, Kadoorie, Hebron, Palestine (App. ID 10-2022). All participants provided informed consent to participate before completing the questionnaire.

## Results

### Demographic characteristics

**Fig. 1:**
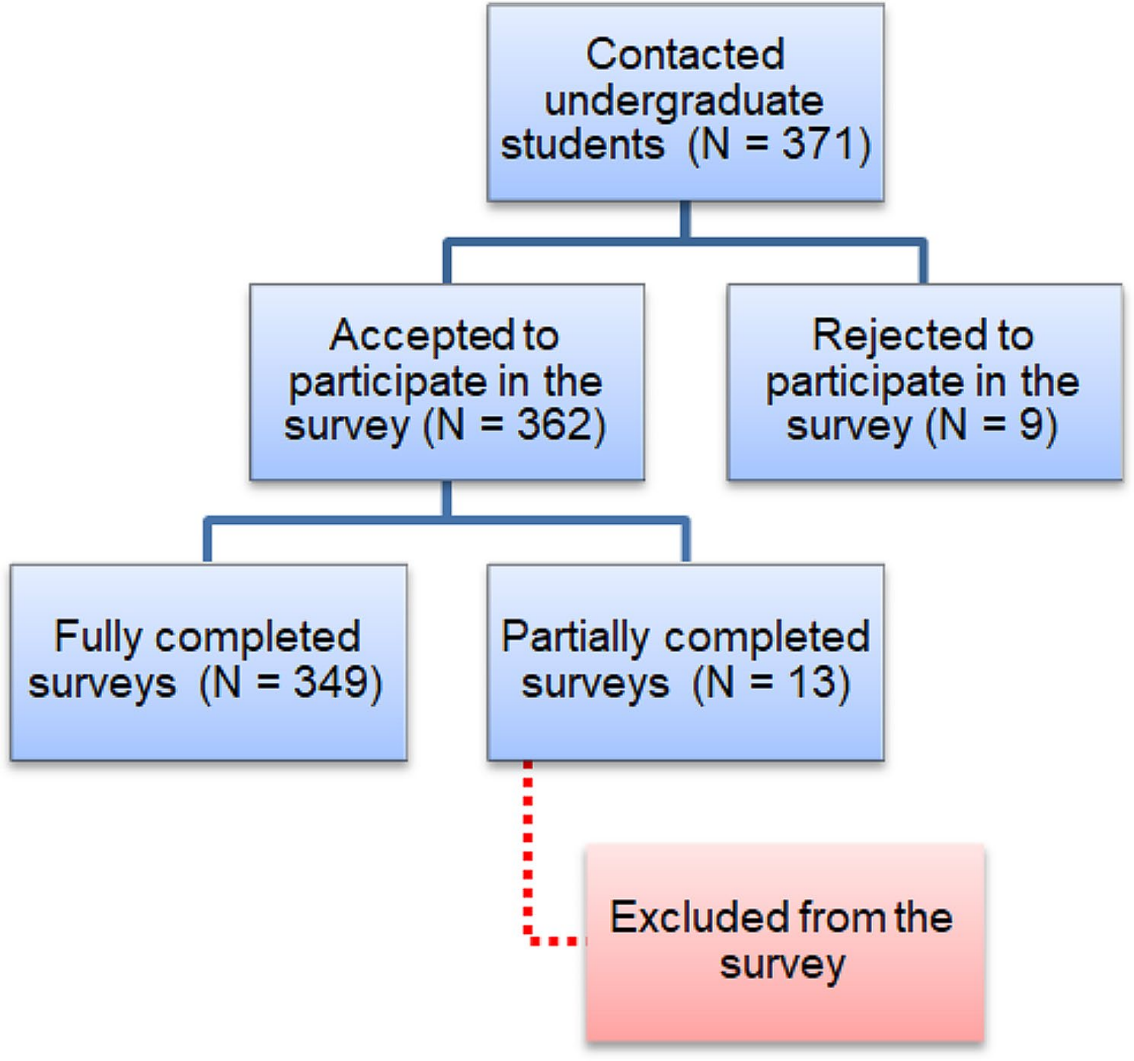
Flow chart presenting the compilation of the study participants

From a total of 371 invitations sent, 362 responses were received (response rate = 97.5%), and 349 were included in the analysis (Fig. 1). Thirteen participants have been excluded from the analysis due to their incomplete responses (partially completed survey). Most of the students (251, 71.92%) were females, and only 28.08% (98) were males. The majority of participants (247, 70.77%) were from the West Bank, with only 29.23% (102) living in the Gaza Strip. Approximately 65.4% (229) of participants were in the age range of 18 to 20 years, and 40.11% (140) were in the second medical school year (M2). In addition, 115 (32.95%) participants confirm that they have a good background in computer science, mathematics, or statistics. 131 (37.54%) participants reported that they have good experience with technology and have acquired a high degree of technological literacy. Only 89 (25.50%) participants reported having a family member with a degree in AI-related majors. The majority of participants were studying medicine at An-Najah National University (85, 24.36%), followed by the Islamic University of Gaza (69, 19.77%), Al-Quds University (57, 16.33%), and Palestine Polytechnic University (52, 14.90%) (Table 2).

**Table 2:** Demographic characteristics of the study participants

Variable		Summary statistics *
Gender		
Female		251(71.92%)
Male		98(28.08%)
Age		
Mean ± Std-Dev		20.38 ± 1.97
Median (Q1-Q3)		20(19–22)
Min, Max		18,26
Age groups		
< 20 years		140(40.11%)
≥ 20 years		209(59.89%)
Year of medical study		
First-year medical school student (M1)		59(16.91%)
Second-year medical school student (M2)		140(40.11%)
Third-year medical school student (M3)		85(24.36%)
Fourth-year medical school student (M4)		31(8.88%)
Fifth-year medical school student (M5)		14(4.01%)
Last-year medical school student (M6)		20(5.73%)
Do you have a background in computer science, mathematics, or statistics?		
No		234(67.05%)
Yes		115(32.95%)
Do you have a good experience in technology or have a high degree of technological literacy?		
No		218(62.46%)
Yes		131(37.54%)
Do you have a parent or sibling with a degree in AI majors?		
No		260(74.50%)
Yes		89(25.50%)
Which university are you currently studying medicine at?		
Gaza Strip	Al-Azhar University	33(9.46%)
	Islamic University of Gaza	69(19.77%)
West Bank	Hebron University	37(10.6%)
	Palestine Polytechnic University	52(14.9%)
	Al-Quds University	57(16.33%)
	An-Najah National University	85(24.36%)
	The Arab American University	16(4.58%)
Total		349 (100%)

*Mean ± SD for continuous variables and n (%) for categorical variables.

### Perceptions about AI in life

The study revealed that 113 (32.38%) participants were aware of the applications of AI in daily life, whereas about two-thirds were not aware (236, 67.62%). Media news (200, 57.31%), literature reviews and published research articles (58, 16.62%), and social media platforms (35, 10.03%) were the most common sources through which students learned about the uses of AI in daily life (Table 3).

Most participants (268, 76.79%) reported that they had not received formal education about AI before or during medical study. During their medical education or training, participants learn about the application of AI in human life from social media platforms (e.g., self-learning groups or YouTube) (120, 34.38%), medical training (48, 13.75%), research projects with research medical teams (46, 13.18%), elective courses provided by the university (45, 12.89%), published research articles (30, 8.6%), or undergraduate curriculum (23, 6.59%).

**Table 3:** Participants’ responses (*n* = 349) to questions related to the use of AI in life

Variable	*N*(%)
I am aware of the applications of AI in our life	
No	236(67.62%)
Yes	113(32.38%)
Where did you hear about the uses and practical applications of AI in our life?	
Lectures at the university	19(5.44%)
Training experience at hospitals	25(7.16%)
Social media platforms	35(10.03%)
Literature reviews and published research articles	58(16.62%)
Media news	200(57.31%)
Colleagues/friends	9(2.58%)
Others	3(0.86%)
Have you had any formal education about AI before or during medical study?	
No	268(76.79%)
Yes	81(23.21%)
Where did you learn about AI during your medical education/training?	
Undergraduate curriculum	23(6.59%)
Elective courses provided by the university	45(12.89%)
Online course	24(6.88%)
Research project with research medical teams	46(13.18%)
Medical training	48(13.75%)
Social media platforms	120(34.38%)
Literature reviews and published research articles	30(8.6%)
Media news	4(1.15%)
Colleagues/friends/professors	4(1.15%)
Scientific events (conferences, workshops, …etc.)	2(0.57%)
Other	3(0.86%)
Do you think that learning programming or mathematics would help you better comprehend the principles and uses of artificial intelligence?	
No	108(30.95%)
Yes	241(69.05%)
Your favorite method of learning about AI in medicine	
Collaborative activities	14(4.01%)
Conferences	29(8.31%)
Extracurricular activities	104(29.8%)
Lectures	92(26.36%)
Workshops	106(30.37%)
Other	4(1.15%)
Total	349 (100%)

In addition, 241 (69.05%) participants thought that learning programming or mathematics would help them better comprehend the principles and uses of AI in medicine. The most highly rated methods of learning about AI in medicine were workshops (106, 30.37%), extracurricular activities (104, 29.8%), lectures (92, 26.36%), as well as participation in scientific activities with other departments (mathematics, computer science) (14, 4.01%). A small proportion of participants (4, 1.15%) confirmed that they preferred other methods to learn about AI, such as in-depth internships with other medical teams and participating in exchange programs with other universities that include AI in their annual course plans.

### Perceptions about AI in medicine

Most participants (250, 71.63%) confirmed knowing that AI, NN, ML, and DL techniques are used in medicine through their responses to the question “Do you know that AI, NN, ML, and DL techniques are used in medicine?“. Social media platforms (119, 34.1%), medical training (47, 13.47%), elective courses provided by the university (46, 13.18%), as well as research projects with research medical teams (45, 12.89%), were the most common sources through which students heard about the uses and applications of AI in medicine (Table 4).

**Table 4:** Participants’ responses (*n* = 349) to questions related to the use of AI in medicine

Do you know that AI, NN, ML, and DL techniques are used in medicine?		
No		99 (28.37%)
Yes		250 (71.63%)
Where did you hear about the employment/integration of AI, NN, ML, and DL in medicine?		
Undergraduate curriculum		23 (6.59%)
Elective courses provided by the university		46(13.18%)
Online course		25(7.16%)
Research project with research medical teams		45(12.89%)
Medical training		47(13.47%)
Social media platforms		119(34.1%)
Literature reviews and published research articles		30(8.6%)
Media news		6(1.72%)
Colleagues/friends/professors		3(0.86%)
Scientific events (conferences, workshops, …etc.)		2(0.57%)
Other		3(0.86%)

ML: machine learning, NN: neural networks, DL: deep learning

**Fig. 2:**
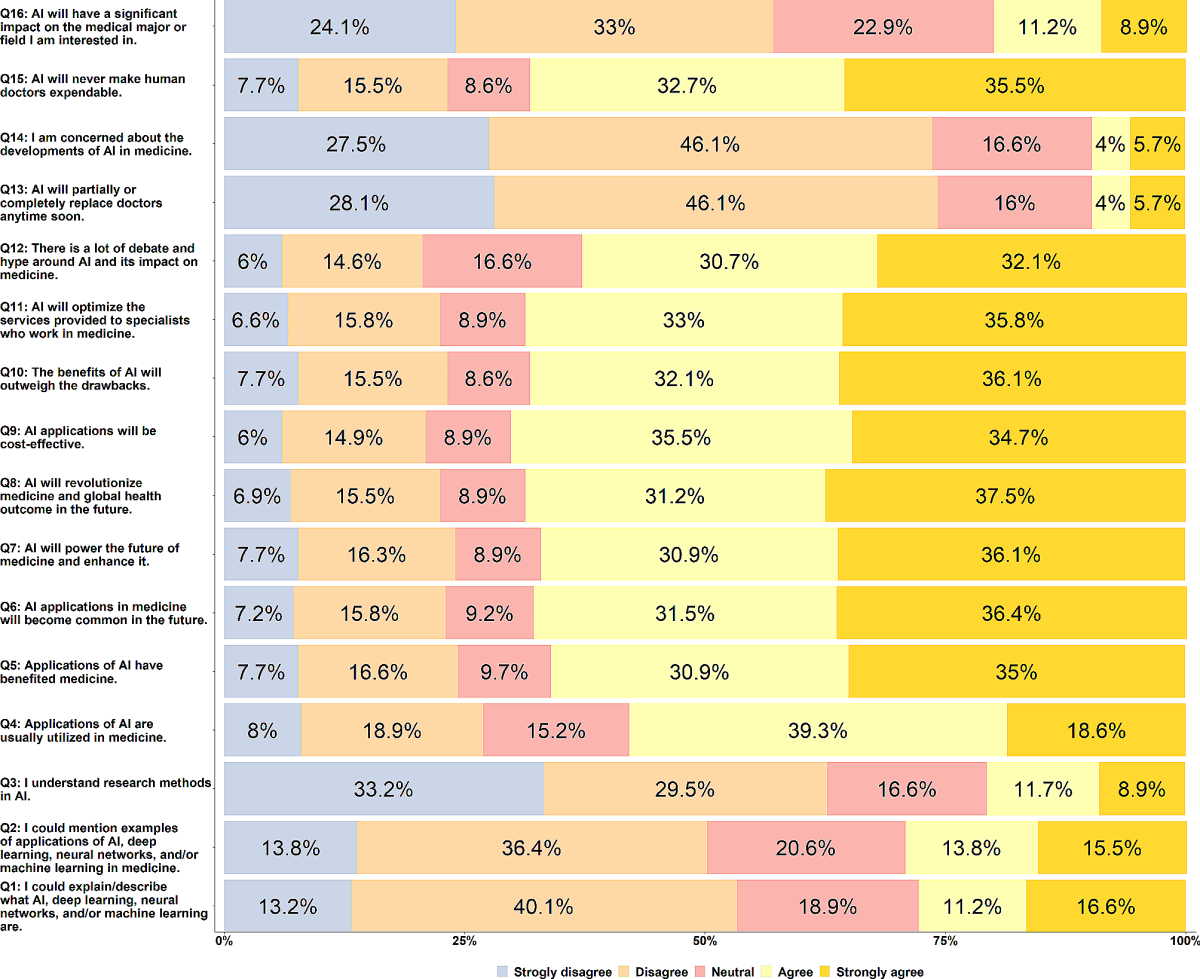
Responses of undergraduate medical students (*n* = 349) to questions related to the future of AI and its current applications in medicine

Figure 2 presents the responses of the study participants to questions related to the future of AI and its current applications in medicine. The overall median score of Likert responses for all participants was 3.38 (IQR = 1). The results showed that only 97 (27.8%) participants strongly agreed or agreed with their ability to understand and describe AI, ML, NN, and DL, and 102 (29.3%) participants believed they could mention different examples of applications of AI, ML, NN, and DL in medicine.

The perceptions of undergraduate medical students of AI in medicine were that it has improved and benefited medicine (65.9%, 230 strongly agree or agree) and that it is usually utilized in medicine (57.9%, 202 strongly agree or agree). About two-thirds of students strongly agreed or agreed that AI would become common in the future (67.9%, 237), power the future of medicine (67.0%, 234), and revolutionize medical fields (68.7%, 240).

Participants strongly agreed or agreed with the idea that AI would be cost-effective (245, 70.2%) and optimize the services provided to specialists who work in medicine (240, 68.8%). Concerning their opinion on whether the benefits of AI would outweigh the drawbacks, 238 (68.2%) strongly agreed or agreed with this statement, 81 (23.2%) strongly disagreed or disagreed, and 30 (8.6%) were not sure. Although there is a lot of debate and hype around AI and its impact on medicine (62.8%, 219), some students did not believe that (72, 20.6%), and others were not sure about this (58, 16.6%).

The study showed that participants did not think that doctors would be completely or partially replaced by AI (259, 74.2% strongly disagree or disagree) and were not concerned about the developments of AI in medicine (257, 73.6% strongly disagree or disagree). Some participants were unsure whether AI would impact their medical-specific major in the future (80, 22.9%). However, more than half (238, 68.2%) of participants agreed with the idea that AI would never make human doctors expendable.

Table 5 illustrates the mean, standard deviation, median, and interquartile range values of the total scores of 5-Likert scale questions related to participants’ perceptions of AI in medicine according to their sociodemographic characteristics. According to Table 5, there was no significant difference between the median scores in terms of gender, age, year of medical study, having a background in mathematics, statistics, or computer science, having a good experience in technology, having a parent or sibling with an AI major, and type of university they are studying at.

**Table 5:** The total score of participants’ perceptions about AI in medicine among different subgroups

variable	*n*(%)	Mean ± SD	Median (IQR)	Max, Min	*P*
**Gender**					
Female	251(71.92%)	3.20 ± 0.75	3.31(1.06)	4.69,1.44	0.22^a^
Male	98(28.08%)	3.31 ± 0.68	3.50(1)	4.69,1.5	
**Age**					
< 20 years	140(40.11%)	2.88 ± 0.81	3.31(0.88)	4.69,1.44	0.78^a^
≥ 20 years	209(59.89%)	2.93 ± 0.91	3.44(1.13)	4.69,1.5	
**Year of medical study**					
M1	59(16.91%)	3.28 ± 0.73	3.38(0.94)	4.5,1.5	0.31^b^
M2	140(40.11%)	3.23 ± 0.72	3.38(0.89)	4.69,1.44	
M3	85(24.36%)	3.09 ± 0.76	3.25(1.19)	4.69,1.5	
M4	31(8.88%)	3.27 ± 0.78	3.31(1.03)	4.38,1.5	
M5	14(4.01%)	3.58 ± 0.63	3.66(0.45)	4.44,1.75	
M6	20(5.73%)	3.37 ± 0.63	3.53(1.11)	4.38,2.38	
**Do you have a background in mathematics, statistics, or computer science?**					
Yes	234(67.05%)	3.21 ± 0.74	3.31(1)	4.69,1.44	0.44^a^
No	115(32.95%)	3.27 ± 0.71	3.44(0.93)	4.69,1.62	
**Do you have a good experience in technology or have a high degree of technological literacy?**					
Yes	218(62.46%)	3.22 ± 0.74	3.31(1)	4.69,1.44	0.70^a^
No	131(37.54%)	3.25 ± 0.71	3.38(0.94)	4.69,1.5	
**Do you have a parent or sibling with a degree regarding AI majors**					
Yes	260(74.5%)	3.21 ± 0.73	3.31(0.96)	4.69,1.44	0.45^a^
No	89(25.5%)	3.28 ± 0.73	3.44(1)	4.69,1.62	
**Which university are you currently studying medicine at?**					
Al-Azhar University	33(9.46%)	3.16 ± 0.7	3.38(0.88)	4.25,1.56	0.18^b^
Islamic University of Gaza	69(19.77%)	3.26 ± 0.75	3.38(0.75)	4.5,1.5	
Hebron University	37(10.6%)	3.32 ± 0.53	3.44(0.5)	4.5,2.12	
Palestine Polytechnic University	52(14.9%)	3.06 ± 0.85	3.09(1.46)	4.69,1.75	
Al-Quds University	57(16.33%)	3.35 ± 0.55	3.44(0.57)	4.56,1.81	
An-Najah National University	85(24.36%)	3.19 ± 0.81	3.31(1.32)	4.38,1.44	
The Arab American University	16(4.58%)	3.40 ± 0.78	3.62(0.67)	4.44,1.5	

a Mann-Whitney U test

b Kruskal-Wallis test

### Perceptions about AI in medical education

**Fig. 3:**
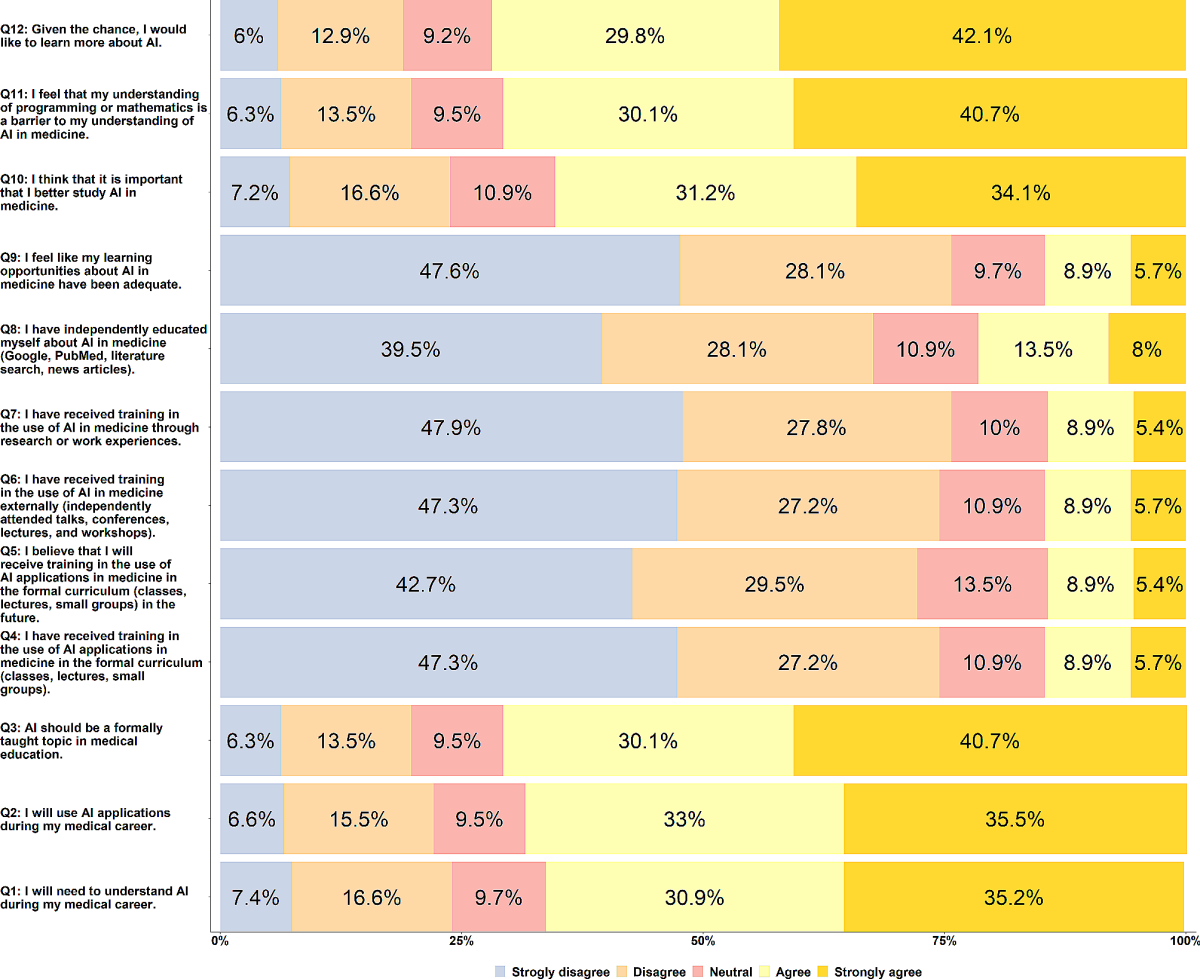
Responses of undergraduate medical students (*n* = 349) to questions related to the Understanding of AI in medical education

Figure 3 illustrates the responses of the study participants to questions related to their understanding of AI in medical education. The overall median score of Likert responses for all participants was 3.00 (IQR = 1). The results showed that participants strongly agreed or agreed (231, 66.1%) that they would need to understand the current uses and applications of AI during their medical career. Approximately two-thirds of medical students (239, 68.5%) confirmed that they would use AI during their medical careers. Participants stated that AI should be specifically included in their medical curriculum (247, 70.8%), but reported that they had never previously acquired training in the use of AI in medicine during formal medical education (260, 74.5%).

Only 14.3% (50) of participants believed that they would receive training regarding employing AI in medicine during formal education in the future. A small percentage of participants confirmed that they received training in the use of AI in medicine outside of formal education (51, 14.6%) (e.g., attending TED talks, scientific conferences, and workshops) or through research and work experience (50, 14.3%). In addition, 75 (21.5%) participants reported that they educated themselves about AI through different scientific websites such as Google, PubMed, literature reviews, and published articles.

**Fig. 4:**
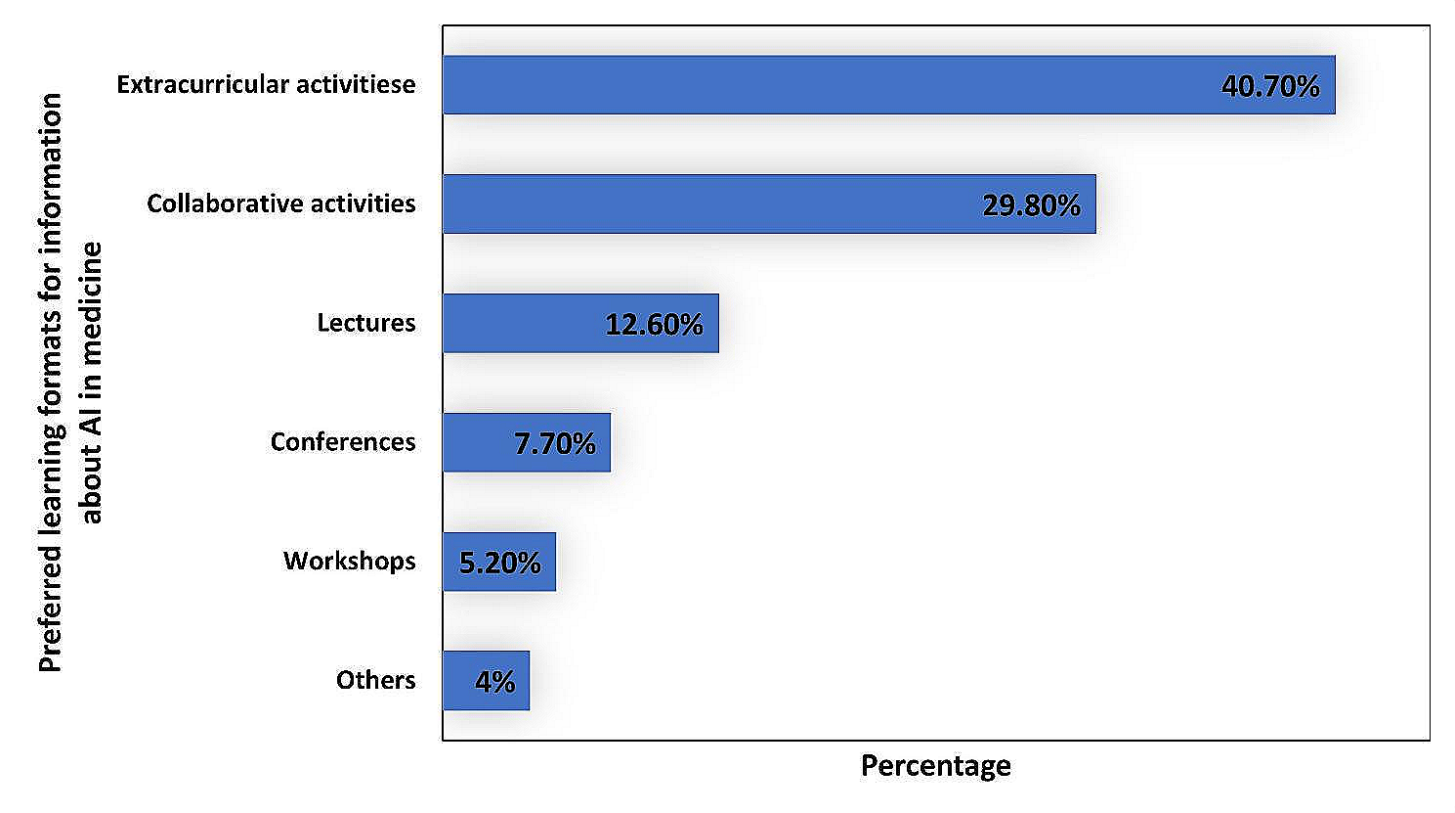
Responses of the study participants (*n* = 349) on the question of the preferred learning formats for information about AI in medicine

Most participants (264, 75.7%) thought that learning opportunities regarding AI in medicine have not been adequate; therefore, it is very important to study more about employing AI in medicine (228, 65.3%). Approximately 70.8% (247) confirmed that the lack of knowledge of mathematics and programming is considered the main problem that hinders understanding AI in medicine. If medical students were given an opportunity, they would learn more about AI (247, 70.8%). Extracurricular activities (142, 40.7%), collaborative activities (104, 29.8%), lectures (44, 12.6%), conferences (27, 7.7%), and workshops (18, 5.2%) were favorite formats for learning and getting more information about AI in medicine (Fig. 4).

Table 6 illustrates the mean, standard deviation, median, and interquartile range values of the total scores of 5-Likert scale questions related to participants’ perceptions of AI in medical education according to their sociodemographic characteristics. A significant difference was found in the mean perception scores concerning gender (*p* < 0.001). Male students (3.15 ± 0.87) had higher perception scores than female students (2.81 ± 0.86). The study found no significant difference in the median scores of participants’ perception of AI in medical education based on age, year of medical study, having a background in mathematics, statistics, or computer science, having a good experience in technology, having a parent or sibling with an AI major, and type of university.

**Table 6:** The total score of participants’ perceptions about AI in medical education among different subgroups

variable	*n*(%)	Mean ± SD	Median (IQR)	Max, Min	*P*
**Gender**					
Female	251(71.92)	2.81 ± 0.86	3(1.17)	5,1	< 0.001^a^
Male	98(28.08%)	3.15 ± 0.87	3(0.98)	5,1	
**Age**					
< 20 years	2.88 ± 0.81	3.22 ± 0.7	3(1)	5,1	0.64^a^
≥ 20 years	2.93 ± 0.91	3.24 ± 0.75	3(1)	5,1	
**Year of medical study**					
M1	59(16.91%)	2.94 ± 0.89	3(1)	5,1	0.36^b^
M2	140(40.11)	2.86 ± 0.79	3(0.6)	5,1	
M3	85(24.36%)	2.79 ± 0.88	3(1.5)	4.75,1.25	
M4	31(8.88%)	2.95 ± 1.02	3(1)	5,1	
M5	14(4.01%)	3.58 ± 1.1	3.42(1.5)	5,1	
M6	20(5.73%)	3.14 ± 0.74	3(1.06)	4.5,2	
**Do you have a background in mathematics, statistics, or computer science?**					
Yes	234(67.05)	2.92 ± 0.87	3(1)	5,1	0.69^a^
No	115(32.95)	2.88 ± 0.89	3(1.04)	5,1	
**Do you have a good experience in technology or have a high degree of technological literacy?**					
Yes	218(62.46)	2.92 ± 0.86	3(1)	5,1	0.86^a^
No	131(37.54)	2.90 ± 0.9	3(1)	5,1	
**Do you have a parent or sibling with a degree regarding AI majors**					
Yes	260(74.5%)	2.89 ± 0.86	3(1)	5,1	0.49^a^
No	89(25.5%)	2.96 ± 0.92	3(1)	5,1	
**Which university are you currently studying medicine at?**					
Al-Azhar University	33(9.46%)	2.99 ± 0.73	3(1)	4.5,1.5	0.75^b^
Islamic University of Gaza	69(19.77%)	3.02 ± 0.82	3(0.92)	5,1	
Hebron University	37(10.6%)	3.02 ± 0.83	3(1)	5,1.5	
Palestine Polytechnic University	52(14.9%)	2.44 ± 0.9	2.5(1.46)	5,1	
Al-Quds University	57(16.33%)	2.86 ± 0.66	3(0.58)	4.5,1	
An-Najah National University	85(24.36%)	3.03 ± 1.02	3(1.66)	5,1	
The Arab American University	16(4.58%)	3.06 ± 0.79	3(1)	4.5,1.5	

a Mann-Whitney U test

b Kruskal-Wallis test

### Interviews with participants

The qualitative analysis of interview questions revealed four main themes: an absence of current AI learning opportunities, the necessity of including AI learning in medical curricula, optimism towards the future of AI in medicine, and expected challenges related to learning AI in medical fields by undergraduate medical students (Table 7).

**Table 7:** Main themes and subthemes resulting from interviews with participants regarding AI in medical education

Main Themes	Subthemes
Current AI learning opportunities	Formal education
	Informal education
Integrate AI learning in medical curricula	Advantages
	Disadvantages
Perceptions towards the future of AI in medicine	Positive perceptions
	Negative perceptions
Expected challenges related to AI	Social challenges
	Economic challenges
	Ethical challenges
	Technological challenges
	Political, policy and legal challenges
	Challenges related to information and data
	Institutional and managerial challenges

### Current AI learning opportunities

The study participants were asked about the current learning opportunities related to AI that were made available to them during or outside of formal education, whether before or after enrollment in medical school.

A high percentage of interviewed participants confirmed that the current learning opportunities regarding AI were informal and outside their medical curricula. They learned about the applications of AI in medicine through different scientific sources such as self-learning from platforms (e.g., edx, Coursera, FutureLearn, and Udemy), participating in international conferences, attending workshops, having in-depth discussions with colleagues, friends, and lecturers, and participating in extracurricular activities with students from other departments (e.g., computer science, mathematics, and information technology). Some students reported that they had a previous opportunity to learn AI through summer camps, internships, and student exchange programs (the Erasmus + Programme).

Below are verbatim undergraduate student interview responses:

Student **A.E.** said that “*I did not get any opportunity during my undergraduate studies related to the use of AI technologies in medicine. Also, the study plan does not include courses related to AI. So my knowledge is very limited in this regard. I wish there was a course on AI in medical fields that would provide us with general information about AI, machine language, and algorithms. I am completely disappointed when I compare myself to students at international universities who have had the opportunity to interact with this topic*”.

Another student, **M.K.**, said that “*I joined the Faculty of Medicine in 2019, and I am now in my fifth year of studying medicine. I can say that I did not have the opportunity to find out what AI is and what its applications are in our lives in general, particularly in medicine. I heard about this term for the first time in an anatomy lecture where the professor who taught us was on a scientific trip to Spain, and he provided us with some information about AI. After that, I started hearing about AI frequently in the news, social media, and scientific journals. I believe that there will be a revolution in the world of medicine if a generation of doctors is prepared to keep pace with modern technological developments*”.

Two students, **J.S.** and **A.R.**, reported that *“I have not taken any training or course related to AI during my formal education at the university. But I heard about this term from my colleague in the Faculty of Engineering, who had the opportunity for an academic exchange (the Erasmus + Programme) at the University of Barcelona in Spain for one semester. When the exchange period ended and he returned to the university, he talked about the differences in the academic system, study plan, courses, workshops, and seminars. My colleague studied* the AI *in Healthcare Specialization course, which highlighted the application of algorithms, machine language, and AI in various medical fields. I hope to have the same opportunity and be part of student exchange programs that enrich students with everything new in the world of technology and medicine*”.

### Integrate AI learning in medical curricula

A small percentage of participants said that including AI in the medical curriculum should not be a top priority, even though the majority of participants thought it was vital. A medical student said that “*I think it is not necessary for medical curricula and study plans to contain courses related to AI because this is one of the tasks of the specialist or expert in the field of AI, machine language, and algorithms. Learning and mastering AI requires great effort and a long time, and this would negatively affect learning the medical skills that I have to master as a doctor.”* On the other hand, some students strongly agreed with the idea of integrating and incorporating the fundamentals of AI into medical curricula. A student stated that “*the curricula should contain courses that provide the student with a good background in the basics of AI without going into the details that are the specialty of the expert”.*

A student stated that “*the fact that the inclusion of AI topics in the courses that a medical student learns is very important in order to enable us to determine a specific path in the future when studying masters and PhDs, such as clinical and genomic diagnostics”*. Also, a student said that “*AI teaches students how to use a diagnostic problem-solving method to address problems relating to diseases. Also, it offers an intelligent simulation tool or surgical aid.”* In addition, he confirmed that “*integrating AI into the medical curriculum can provide better learning by identifying students’ comprehension levels and serving as a tool for clinical decision-making*”.

Three students in their final year of medical school agreed that there is a need to be self-reliant and to build partnerships and relationships with medical students at other universities virtually in order to fill knowledge gaps related to employing AI techniques in medicine. They said that “*from my personal experience, I have joined medical groups that publish all updates in the field of medicine, including the importance of* AI *in diagnosis, treatment, data analysis, and chemical examinations. I knew about the FutureLearn platform, which offers a free course entitled “Digital Skills: Artificial Intelligence,” which was accredited by the CPD Certification Service. The duration of the course was 3 weeks, and I learned a general background on* AI *(1st week)*, AI *in the industry (2nd week), and adapting our skills to work with* AI *(3rd week)*”.

Student **N.W.** said that “*it is true that the formal curriculum that we studied at the university lacks programming, machine learning, and AI. This does not mean that the medical student should stay without doing anything. He must research, study, and teach himself. Enrolling in training courses related to AI contributes to improving the individual’s level of awareness of the need to employ them in the future. Engaging in scientific conferences, workshops, and internships contributes to mitigating the severity of illiteracy that we suffer from*”.

Another medical student in their 4th year of study said that “*technological developments in the medical field must be kept updated because we live in Gaza and the economic situation in general is deteriorating, so it is difficult for a medical student to participate in any scientific event as long as there is no funding. If I have the opportunity to obtain funding from a donor, I will definitely take intensive training related to applying AI tools in my major and engaging with my peers from other countries”*.

#### Perceptions towards the future of AI in medicine

Participants revealed a variety of emotions towards AI. Some reported their optimism for the future of technology, while others voiced concerns that it would be misused. Some students indicated that AI would be very useful in medicine in terms of diagnosis, analysis, characterization, calculating the number of chemicals required, online appointment scheduling, performing surgical operations with high accuracy, and detecting drug dosage.

A student, **A.S.**, stated that “*despite the presence of some concerns, there are numerous applications of AI in medical and healthcare fields, such as online check-in at medical facilities, digitization of medical records, reminder calls for follow-up appointments, and immunization dates for children and pregnant women*”.

Also, a student who joined an AI course on the edx platform reported that “*I just finished registering for a new course entitled AI in Practice: Applying AI, which will be provided by the Delft University of Technology on the edx platform. I will learn how to build a step-by-step plan for implementing AI in my organization, as well as the implementation and practical aspects of AI. I am very excited to start the course next September, and I expect that I will be able to learn how to improve the medical field using AI*”.

On the other hand, student **J.S.** referred to “*Despite the importance of AI tools in the medical field, there are ethical concerns that have been raised by a large number of critics, and the controversy still exists.”* Similarly, another student stated that “*if an error is detected in the AI tools used, this error will be catastrophic. Although the AI tools are accurate, errors can occur.”* In addition, a student confirmed that “*I think that clinicians will worry about job security. There will be fewer positions for doctors in specific fields that require AI, where AI will be more applicable than human doctors, such as pathology, radiology, cardiology*, *dermatology, and ophthalmology.”* Also, a student said that “*Even though most patients are inclined to accept an AI-based diagnosis, they tend to trust doctors more often when their diagnosis disagrees with the AI’s. I think that only a small number of medical students have access to personal-level data science or machine learning courses, despite their need for such training. Additionally, doctors may worry a lot about being replaced in the future by AI.”* Some students raised the problem of data privacy and security, as AI in medicine needs large amounts of data from patients. Some students agreed that “a *patient’s right to determine how their data is used and protection from unwanted access to that data are both crucial*”.

### Expected challenges related to learning AI

Questions were asked of the students who were interviewed about the challenges, difficulties, and obstacles they expected to face if AI were to be implemented in medical fields in the future. The obstacles mentioned by the students included the obstacles they expected to face when studying AI courses during their formal education or the obstacles they would face when they practically employed AI in medicine during their medical careers. The participants mentioned social, economic, ethical, technological, political, institutional, and information challenges.

A student said that “*when the Faculty of Medicine decides to include AI courses in the study plan, I expect that we will face many difficulties related to adapting to these subjects and how to employ them in the field of medicine. In addition, most students suffer from weaknesses in mathematics, statistics, and computer science, which hinder their understanding of the principles and basics of AI*. *I believe that one of the main obstacles to the adoption of AI in medicine is cultural, as some undergraduate medical students may be reluctant to engage with novel devices and programs*”.

Another student stated that “*from my point of view, studying AI courses may hinder the comprehension and understanding of other courses related to medicine. For example, I need 3 hours a day to study the AI course, and this reduces the time required to study medical courses such as anatomy and physiology. This means that I will waste time studying topics that have no practical application in my country”*.

Similarly, student **K.S.** reported that “*mastering the principles of statistics, mathematics, programming, and algorithms is very important before starting to focus on studying AI. The student of the Faculty of Medicine did not receive sufficient education during secondary school, so he faced many difficulties and challenges when transferring from school to university. Therefore, medical students should master the basics before moving on to studying deep topics*”.

Another medical student in his second year of medical school confirmed that “*Since we live in Gaza, the continuous cut of electricity and internet, the lack of logistical services, and the scarcity of specialists in AI are among the biggest challenges that prevent understanding this field. In addition, the deteriorating economic situation will play a major role in preventing the student from participating in related courses, workshops, and lectures outside of formal education, as the medical student hardly provides tuition fees and monthly expenses.”* Then, he concluded, “*Given the required investment and altered working procedures, the widespread adoption of AI technology could have a large negative economic impact on organizations and institutions”*.

A student in his third year of medical school reported that “*through my journey in preparing educational material about AI in medicine for high school students, I read research papers about the difficulties of AI and big data integration. New and effective solutions are required to handle big data’s enormous volume.”* Moreover, some students raised challenges related to institutions, which would depend on AI in the future. A student mentioned that “organizations may suffer serious problems when there is no plan in place for how *AI can disrupt important business sectors and fail to address issues in the human workforce”*.

Misunderstanding and lack of knowledge are factors that contribute to the expansion of the gap and complicate the situation among undergraduate medical students. For example, a medical student reported that “*many medical students may be unaware of how AI functions and what it can and cannot achieve. This may cause medical students to have irrational expectations and to lose faith in technology*. *Also, a barrier to incorporating AI in our curriculum is the priority given to non-AI medical subjects*”.

A student who joined an online course about AI on the Udemy platform clarified that some learners discussed legal problems related to AI. He quoted a comment written within the group: “*There are many legal issues surrounding AI for mistakes made by this system. The problem of copyrights may present another legal challenge when deploying AI systems. To adequately safeguard and reward human-generated work, the current legal system has to undergo considerable adjustments*”.

## Discussion

The results of the study revealed that most participants thought that AI was very important to medical fields and urgently wanted opportunities to learn the fundamentals of AI. The study showed that there are still not enough educational options available in Palestine at the universities that teach medicine. The incorporation of learning and training opportunities about AI in the traditional medical curriculum needs to be taken into consideration given the quick advancement and the increasing usage of AI in the medical fields around the world. Additionally, as the skills needed to use AI may differ from those traditionally possessed by medical students, the integration of AI content in medical curricula must take into account the preferred learning formats, learning content, and expected challenges that may be faced by medical students.

The results of this study are in agreement with the study of Teng et al. [46], who explored the perspectives of healthcare students on AI in Canada. They found that the participants had limited AI-related knowledge. According to a 2020 European survey, only one-third of medical students said they knew the basics of AI [41]. According to a survey conducted in 2021 among medical students in Ontario, participants thought they grasped the meaning of AI, but when asked about specialized terminologies related to AI (such as ML, NN, etc.), they were unable to understand them [28]. Similar findings were also reported in other studies involving healthcare and medical students in Germany [25, 29], Pakistan [31], Syria [37], India [47], and Vietnam [48], where the medical students have insufficient knowledge of AI. More recently, a review conducted by Mousavi Baigi et al. [49] showed that most medical students had little knowledge of and limited skills in working with AI, indicating that immediate education is necessary in this regard. This expanding knowledge gap could pose a challenge to the effective incorporation of AI in medicine [50]. On the other hand, some studies found adequate information and a high level of knowledge about AI among medical students [36, 49].

Currently, most medical schools do not have a formal AI curriculum [51]. Therefore, medical students lack both knowledge and skill. Some studies state that many students claim never to have heard of machine learning, and their medical school taught them little to nothing about AI [52, 53]. Most medical students are willing to learn more about AI, but many face obstacles to understanding the statistical techniques employed in AI research, prospective applications, or interpreting the results of AI-related publications [54].

Contrary to the uncertainty about AI applicability, some participants were positive about AI in their medical disciplines [46]. We also observed some inconsistencies among the study participants. Some thought AI would revolutionize medicine but also thought it would not directly impact their medical careers. These results can be attributed to several reasons, such as the lack of knowledge of AI applications, misconceptions around AI perpetuated by the media, and a lack of practical exposure to AI in a medical environment. Similarly, a study conducted on medical physics students revealed that most students believed that AI would revolutionize medicine and enhance healthcare and disagreed that AI threatened their careers in the future [55].

Our results revealed that most participants did not think that doctors would be replaced by AI and were not concerned about the developments of AI in medicine. These results are inconsistent with the study carried out by Pinto dos Santos et al. [25], which indicated that this issue was not a concern for the students. On the other hand, a previous study conducted with medical students showed that the participants developed concerns about the possibility of AI replacing human doctors in the upcoming years [56]. Therefore, this concern has made students less likely to select imaging-based diagnostic majors like radiology. Even though concerns about AI may differ depending on the study groups, incorporating AI into medical curricula may mitigate their worries about its application in a medical environment. In addition, it was reported that a lack of understanding may make it difficult for doctors to deal with AI technology effectively as it becomes more prevalent in hospitals [46]. Therefore, incorporating AI into medical curricula may resolve this issue. To fill this expanding gap in knowledge, medical curricula must be improved in light of current advancements in the AI field [29, 40, 46].

The present study presents an evaluation of current AI learning opportunities, preferred AI education delivery forms, and expected challenges. Our study revealed that there are no universities that have a formal AI program included in the annual plan. This is due to the many challenges that Palestinian universities face, the most important of which is the lack of funding and the absence of material and technical support. Technological illiteracy plays an important role in this problem, as there is a general weakness in the basics of computer science, mathematics, and programming. This situation requires teaching medical students these principles before incorporating AI into the medical curriculum, and this in turn requires a longer time (two semesters), which may be rejected by students and universities. To avoid exacerbating the problem in the future, universities should focus on other strategies that gradually build the knowledge of medical students about AI. Summer training courses and extracurricular activities can be offered, particularly for those who are interested. Similar situations have been previously observed in other countries [51, 57, 58], where opportunities for learning about AI in medical education are very limited. In addition, it was reported that medical students lack structured education related to AI, which can make them feel unprepared and ignorant. Therefore, designing AI courses is critical to ensuring that medical students have the skills they need to succeed in their future medical professions [59].

Our study revealed that male students had higher perception scores than female students. This result may be attributed to the fact that male students have more information and adequate knowledge related to AI than female students [48, 60]. Also, students who join workshops and attend courses on AI in medicine will become educated and have more knowledge of AI; therefore, they will have a higher perception and awareness of AI-related topics. Similar results were found among medical students in Turkey, where males had higher mean PAIM (Perceptions on Artificial Intelligence in Medicine) scores than females [42]. A previous study conducted by Sarwar et al. [61] on physician perspectives on the integration of AI into diagnostic pathology found that males were more likely to integrate AI, had a more positive attitude toward the application of AI in practice, and felt more at ease using computer science technologies and AI tools than females. Moreover, a study carried out among elementary students in China showed that male students felt more confident and had higher readiness for AI compared to female students after attending an AI course [62].

The interviews conducted with participants highlighted different challenges and difficulties they expected to face when studying AI courses during their formal education or when implementing AI in medicine during their medical career if AI is incorporated into medical fields. The participants reported social, economic, ethical, technological, political, institutional, and information challenges. Similar results have been previously discussed in the study of Grunhut et al. [63]. They revealed that medical schools do not incorporate AI into their curricula for several reasons, such as a lack of faculty expertise, a lack of evidence to support students’ growing interest in AI-related courses, and the absence of recommendations from the responsible committees of medical education. Many challenges play key roles in deciding whether AI will be incorporated into medical curricula or not, such as the experience of faculty members, having data about backup students’ growing interest in learning about AI, or the presence of recommendations from responsible authorities on medical education [64, 65]. Therefore, the diversity of AI tools, the methodology for engineering and building AI solutions to medical problems, and the significance of data in the creation of AI advances should all be understood by medical students to mitigate possible difficulties.

During the interviews, we noticed that one barrier to incorporating AI is the priority given by the students to non-AI medical subjects. The utilization of unique educational methods could help overcome this challenge since the study participants indicated that workshops, collaborative activities, scientific conferences, and extracurricular activities were their favorite learning types. These would be simpler to apply in a complex medical curriculum and more agreeable to medical students [66]. Although some participants expressed concern that a lack of mathematical or computer science understanding would limit effective learning about AI, the rest did not believe that technical knowledge would be a challenge to the understanding of AI. Given the wide range of educational experiences and technological knowledge, it is wise for medical AI curricula to refrain from delving into intricate technical details. To attract the attention of the students, it should design AI materials without rigorous mathematics, address the concern about AI, provide students with open access resources, and build teams with multidisciplinary collaboration [67].

Numerous studies have shown that there is a key problem with the understanding of AI and that medical curricula should address AI and include it as the main learning content. AI curriculum building can be achieved in a well-informed manner in Palestine due to insights provided by this study and results regarding preferred AI education formats and obstacles to AI education. We recommend providing Palestinian medical students with AI subjects according to the attractive formats they prefer, such as workshops, lectures, extracurricular activities, and conferences. We also advised planning to educate medical students during summer vacation, as they have time to learn and study. Medical education must quickly shift from the information age to the age of AI to avoid the problem of leaving medical students unprepared to use AI in clinical environments [68].

Educational initiatives that aim to educate undergraduate medical students in the AI field should be adopted and given priority by the responsible authorities. We strongly recommend conducting studies focused on developing instructional materials in the aforementioned formats and evaluating them with a group of medical students in Palestine.

## Conclusion

This study highlights the attitudes of medical students toward AI in medicine, training opportunities, and preferred methods for teaching AI curricula. Most participants did not receive formal education on AI before or during medical study and suffered from a severe lack of theoretical and/or practical educational opportunities related to the use of AI techniques in medicine. As the participants had not previously acquired training in the employment of AI in medicine during formal education, this confirmed a dire need for the incorporation of AI courses in medical curricula. The incorporation of AI in medical education, the creation of training environments, and equipping students with the necessary skills should be taken into account to support their learning, providing personalized experiences and improved outcomes. Given that AI instruments are likely to be extensively utilized in the future, training the next generation of medical students in how AI will fit into clinical practices will help them make major changes in the medical field, enhancing the careful use of these instruments in manual practice and ultimately improving the healthcare system.

### Strengths and limitations

The strengths of the present research include its temporal proximity to technological developments regarding the integration of AI in medical education around the world. This study is considered the first of its kind conducted in Palestine. Based on our knowledge, no previous Palestinian study focused on the perceptions of medical students about AI in medicine. Therefore, the results should be taken into consideration to improve medical curricula and adopt new strategies for educational content. Limitations of this study include the non-representativeness of the study sample, which mainly consisted of female students (71.92%). This may limit the generalizability of our findings to a wider population. The second limitation is present in the fact that our study was conducted at only seven universities in Palestine that have a faculty of medicine. Due to self-selection bias caused by voluntary participation, only medical students interested in AI and medical technologies probably completed the survey. This study’s sample size is not representative of all governorates of Palestine, where most of the participants were from the West Bank. Finally, this study lacked sensitivity analyses. In our study to address self-selection bias, we followed some techniques such as using multiple methods of data collection (online surveys and face-to-face interviews), anonymity and confidentiality of the participant’s responses, conducting pilot testing of the survey instrument, following ethical guidelines, and consider accessibility features for the study online survey.

### Electronic supplementary material

Below is the link to the electronic supplementary material.

Supplementary Material 1

Supplementary Material 2

Supplementary Material 3

Supplementary Material 4

## Data Availability

The data presented in this study are available on request from the corresponding author.

## Abbreviations

AI: Artificial intelligence
ML: Machine Learning
NN: Neural Networks
DL: Deep Learning
