# Supplementary material for: Perceptions of undergraduate medical students on artificial intelligence in medicine: mixed-methods survey study from Palestine
Source: BMC Med Educ. 2024 May 7;24:507. doi: 10.1186/s12909-024-05465-4 (PMC11077786; doi:10.1186/s12909-024-05465-4)
Supplement: Supplementary file 2 — Supplementary Material 2 [file 12909_2024_5465_MOESM2_ESM.docx]

**Perceptions of undergraduate medical students on artificial intelligence in medicine: mixed-methods survey study from Palestine**

**Dear Participants,**

Thank you for participating in this study. Your feedback is appreciated and extremely valued. The purpose of this study is to identify undergraduate medical students' attitudes toward AI in medicine, explore present AI-related training opportunities, investigate the need for AI inclusion in medical curricula, and determine preferred methods for teaching AI curricula.

Participation is voluntary, you can withdraw at any time. All answers will be kept confidential. Filling out the survey will be construed as consent. The survey should not take more than 8-10 minutes.

Kindly, check the answer category that most describes you. Your time and effort are greatly appreciated.

**This survey consists of 5 sections:**

**1) Screening and Logistics;**

**2) Demographics;**

**3) AI in Daily Life;**

**4) Artificial Intelligence in Medicine;**

**5) Artificial Intelligence in Medical Education.**

**Section 1. Screening and Logistics**

| - 1. Do you consent to participate in this survey? |
| --- |
| Yes |
| No |
| - 1. Are you currently a Palestinian medical student and enrolled in the first semester 2022-2023? |
| Yes |
| No |
| - 1. Would you be willing to provide a short, recorded interview about your responses at a later date? |
| Yes |
| No |
| - 1. If yes, Please provide your email address. It will be used to filter out spam responses, enable withdrawal, and contact potential interviews. No identifiable data will ever be published or made publicly available. |
| Email address:………………………………………………………………………... |

**Section 2. Demographic characteristics**

| 2.1. Gender |
| --- |
| Female |
| Male |
| 2.2. Age |
| 19 years |
| 20 years |
| 21 years |
| 22 years |
| 23 years |
| 24 years |
| More than 25 years |
| 2.3. Year of medical study |
| First-Year Medical School Student (M1) |
| Second-Year Medical School Student (M2) |
| Third-Year Medical School Student (M3) |
| Fourth-Year Medical School Student (M4) |
| Fifth-Year Medical School Student (M5) |
| Last-Year Medical School Student (M6) |
| 2.4. Do you have a background in mathematics, statistics, or computer science? |
| Yes |
| No |
| 2.5. Do you have a good experience in technology or have a high degree of technological literacy? |
| Yes |
| No |
| 2.6. Do you have a parent or sibling with an a degree regarding AI majors |
| Yes |
| No |
| 2.7. Which university are you currently studying medicine at? |
| Al-Azhar University |
| Islamic University of Gaza |
| Hebron University |
| Palestine Polytechnic University |
| Al-Quds University |
| An-Najah National University |
| The Arab American University |

**Section 3. AI in Daily Life**

| 3.1. I am aware of the applications of AI in our life |
| --- |
| Yes |
| No |
| 3.2. Where did you hear about the uses and practical applications of AI in our life? |
| Lectures at the university |
| Training experience at hospitals |
| Social media platforms |
| Literature reviews and published research articles |
| Media news |
| Colleagues/friends |
| Others |
| 3.3. Have you had any formal education about AI before or during medical study? |
| Yes |
| No |
| 3.4. Where did you learn about AI during your medical education/training? |
| Undergraduate curriculum |
| Elective courses provided by the university |
| Online course |
| Research project with research medical teams |
| Medical training |
| Social media platforms |
| Literature reviews and published research articles |
| Media news |
| Colleagues/friends/professors |
| Scientific events (conferences, workshops, …etc.) |
| Other |
| 3.5. Do you think that learning programming or mathematics would help you better comprehend the principles and uses of artificial intelligence? |
| Yes |
| No |
| 3.6. Your favorite method of learning about AI in medicine |
| Lectures |
| Workshops |
| Conferences |
| Extracurricular activities |
| Collaborative activities with other departments (mathematics, computer science |
| Other |

**Section 4. AI in medicine**

| 4.1. Do you know that AI, neural networks, machine learning, and deep learning techniques are used in medicine? | | | | |  |
| --- | --- | --- | --- | --- | --- |
| Yes | | | | |  |
| No | | | | |  |
| 4.2. Where did you hear about the employment/integration of AI, neural networks, machine learning, and deep learning in medicine? | | | | |  |
| Undergraduate curriculum | | | | |  |
| Elective courses provided by the university | | | | |  |
| Online course | | | | |  |
| Research project with research medical teams | | | | |  |
| Medical training | | | | |  |
| Social media platforms | | | | |  |
| Literature reviews and published research articles | | | | |  |
| Media news | | | | |  |
| Colleagues/friends/professors | | | | |  |
| Scientific events (conferences, workshops, …etc.) | | | | |  |
| Other | | | | |  |
| 4.3. I could explain/describe what AI, deep learning, neural networks, and/or machine learning are. | | | | | |
| Strongly disagree | Disagree | Neutral | Strongly agree | Agree | |
| 4.4. I could mention examples of applications of AI, deep learning, neural networks, and/or machine learning in medicine. | | | | | |
| Strongly disagree | Disagree | Neutral | Strongly agree | Agree | |
| 4.5. I understand research methods in AI | | | | | |
| Strongly disagree | Disagree | Neutral | Strongly agree | Agree | |
| 4.6. Applications of AI are usually utilized in medicine. | | | | | |
| Strongly disagree | Disagree | Neutral | Strongly agree | Agree | |
| 4.7. Applications of AI have benefited medicine. | | | | | |
| Strongly disagree | Disagree | Neutral | Strongly agree | Agree | |
| 4.8. AI applications in medicine will become common in the future. | | | | | |
| Strongly disagree | Disagree | Neutral | Strongly agree | Agree | |
| 4.9. AI will power the future of medicine and enhance it. | | | | | |
| Strongly disagree | Disagree | Neutral | Strongly agree | Agree | |
| 4.10. AI will revolutionize medicine and global health outcomes in the future. | | | | | |
| Strongly disagree | Disagree | Neutral | Strongly agree | Agree | |
| 4.11. AI applications will be cost-effective | | | | | |
| Strongly disagree | Disagree | Neutral | Strongly agree | Agree | |
| 4.12. The benefits of AI will outweigh the drawbacks. | | | | | |
| Strongly disagree | Disagree | Neutral | Strongly agree | Agree | |
| 4.13. AI will optimize the services provided to specialists who work in medicine. | | | | | |
| Strongly disagree | Disagree | Neutral | Strongly agree | Agree | |
| 4.14. There is a lot of debate and hype around AI and its impact on medicine. | | | | | |
| Strongly disagree | Disagree | Neutral | Strongly agree | Agree | |
| 4.15. AI will partially or completely replace doctors anytime soon. | | | | | |
| Strongly disagree | Disagree | Neutral | Strongly agree | Agree | |
| 4.16. I am concerned about the developments of AI in medicine. | | | | | |
| Strongly disagree | Disagree | Neutral | Strongly agree | Agree | |
| 4.17. AI will never make human doctors expendable. | | | | | |
| Strongly disagree | Disagree | Neutral | Strongly agree | Agree | |
| 4.18. AI will have a significant impact on the medical major or field I am interested in. | | | | | |
| Strongly disagree | Disagree | Neutral | Strongly agree | Agree | |
| 4.19. If you associate artificial intelligence applications with a specific discipline in medicine, which ones (fill as many as appropriate)? | | | | | |
| Anatomical pathology | | | | | |
| Anesthesiology | | | | | |
| Cardiac surgery | | | | | |
| Dermatology | | | | | |
| Radiology | | | | | |
| Emergency medicine | | | | | |
| Family medicine | | | | | |
| Family medicine – Integrated care of the elderly | | | | | |
| Family medicine – Integrated emergency medicine | | | | | |
| General pathology | | | | | |
| General surgery | | | | | |
| Hematological pathology | | | | | |
| Internal medicine | | | | | |
| Medical genetics and genomics | | | | | |
| Medical microbiology | | | | | |
| Neurology | | | | | |
| Neurology – Pediatric | | | | | |
| Neuropathology | | | | | |
| Neurosurgery | | | | | |
| Nuclear medicine | | | | | |
| Obstetrics and gynecology | | | | | |
| Otolaryngology – Head and neck surgery | | | | | |
| Pediatrics | | | | | |
| Physical medicine and rehabilitation | | | | | |
| Plastic surgery | | | | | |
| Psychiatry | | | | | |
| Public health and preventative medicine | | | | | |
| Public health and preventative medicine including family medicine | | | | | |
| Radiation oncology | | | | | |
| Urology | | | | | |
| Other | | | | | |
| Vascular surgery | | | | | |
| Other | | | | | |

**Section 5. AI in medical education**

| 5.1. I will need to understand AI during my medical career. | | | | |
| --- | --- | --- | --- | --- |
| Strongly disagree | Disagree | Neutral | Strongly agree | Agree |
| 5.2. I will use AI applications during my medical career. | | | | |
| Strongly disagree | Disagree | Neutral | Strongly agree | Agree |
| 5.3. AI should be a formally taught topic in medical education. | | | | |
| Strongly disagree | Disagree | Neutral | Strongly agree | Agree |
| 5.4. I have received training in the use of AI applications in medicine in the formal curriculum (classes, lectures, small groups). | | | | |
| Strongly disagree | Disagree | Neutral | Strongly agree | Agree |
| 5.5. I believe that I will receive training in the use of AI applications in medicine in the formal curriculum (classes, lectures, small groups) in the future. | | | | |
| Strongly disagree | Disagree | Neutral | Strongly agree | Agree |
| 5.6. I have received training in the use of AI in medicine externally (independently attended talks, conferences, lectures, and workshops). | | | | |
| Strongly disagree | Disagree | Neutral | Strongly agree | Agree |
| 5.7. I have received training in the use of AI in medicine through research or work experiences. | | | | |
| Strongly disagree | Disagree | Neutral | Strongly agree | Agree |
| 5.8. I have independently educated myself about AI in medicine (Google, PubMed, literature search, news articles). | | | | |
| Strongly disagree | Disagree | Neutral | Strongly agree | Agree |
| 5.9. I feel like my learning opportunities about AI in medicine have been adequate. | | | | |
| Strongly disagree | Disagree | Neutral | Strongly agree | Agree |
| 5.10. I think that it is important that I better study AI in medicine. | | | | |
| Strongly disagree | Disagree | Neutral | Strongly agree | Agree |
| 5.11. I feel that my understanding of programming or mathematics is a barrier to my understanding of AI in medicine. | | | | |
| Strongly disagree | Disagree | Neutral | Strongly agree | Agree |
| 5.12. Given the chance, I would like to learn more about AI. | | | | |
| Strongly disagree | Disagree | Neutral | Strongly agree | Agree |
| 5.13. My preferred format for learning about artificial intelligence and machine learning in medicine are (fill as many as appropriate) | | | | |
| Lectures | | | | |
| Conferences | | | | |
| Workshops | | | | |
| Extracurricular activities | | | | |
| Collaborative activities with other departments (mathematics, computer science) | | | | |
| Other | | | | |
