# Supplementary material for: Perceptions of undergraduate medical students on artificial intelligence in medicine: mixed-methods survey study from Palestine
Source: BMC Med Educ. 2024 May 7;24:507. doi: 10.1186/s12909-024-05465-4 (PMC11077786; doi:10.1186/s12909-024-05465-4)
Supplement: Supplementary file 3 — Supplementary Material 3 [file 12909_2024_5465_MOESM3_ESM.docx]

**Interview questions**

1. What year of medical school are you now in? Also, please let us know about your age and academic background.
2. What are your knowledge of artificial intelligence in medicine?
3. Do you believe that learning about artificial intelligence is important throughout medical school?
4. Do you believe AI will be crucial to your practice as a doctor in the future?
5. What opportunities have you had to learn about artificial intelligence in medicine whether formal or informal curriculum?
6. What learning opportunities to learn about artificial intelligence would you like to have in the future?
7. Is there anything else you would like to share about artificial intelligence in medicine or anything related to this topic?
